# Perfluorooctane sulfonate affects intestinal immunity against bacterial infection

**DOI:** 10.1038/s41598-017-04091-z

**Published:** 2017-07-12

**Authors:** Caixia Suo, Zhiqin Fan, Liang Zhou, Ju Qiu

**Affiliations:** 1 The Key Laboratory of Stem Cell Biology, Institute of Health Sciences, Shanghai Institutes for Biological Sciences, Chinese Academy of Sciences and Shanghai Jiao Tong University School of Medicine, University of Chinese Academy of Sciences, Shanghai, 200031 China; 2 Department of Infectious Diseases and Pathology, College of Veterinary Medicine The University of Florida, Gainesville, FL 32608 USA

## Abstract

Perfluorooctane sulfonate (PFOS) is an environmental contaminant that has been manufactured to be used as surfactants and repellents in industry. Due to long half-life for clearance and degradation, PFOS is accumulative in human body and has potential threat to human health. Previous studies have shown the development and function of immune cells can be affected by PFOS. Although PFOS has a high chance of being absorbed through the oral route, whether and how PFOS affects immune cells in the gut is unknown. Using mouse model of *Citrobacter rodentium* infection, we investigated the role of PFOS on intestinal immunity. We found at early phase of the infection, PFOS inhibited the expansion of the pathogen by promoting IL-22 production from the group 3 innate lymphoid cell (ILC3) in an aryl hydrocarbon receptor dependent manner. Nevertheless, persistent PFOS treatment in mice finally led to a failure to clear the pathogen completely. At late phase of infection, enhanced bacterial counts in PFOS treated mice were accompanied by increased inflammatory cytokines, reduced mucin production and dysbiosis, featured by decreased level of *Lactobacillus casei*, *Lactobacillus johnsonii* and increased *E. coli*. Our study reveals a deleterious consequence in intestinal bacterial infection caused by PFOS accumulation.

## Introduction

Perfluorooctane sulfonate (PFOS) is widely used as surface treatment chemical, polymerization acid and surfactant in industry, due to its chemical stability, high surface activity and water and oil repellence property. The 3 M Company, main manufacturer of PFOS, phased out the product in 2002 because of toxic effects of PFOS to the human endocrine, reproductive and immune systems^1–4^. In 2009, the 4^th^ meeting of the Conference of the Parties to the Stockholm Convention listed PFOS to the Annex B to limit the use of PFOS^5^. Though the application of PFOS has been abandoned by its main manufacturer, emission of PFOS still persists in many countries due to a lack of cost-efficient alternatives^6, 7^. Besides, the half-life for clearance of PFOS in the serum is as long as 4.8 years on average^8^. Thus, PFOS is bio-accumulative in water and ground and remains to be a potential threat to human health worldwide.

PFOS has been reported to affect the immune cells in the central and peripheral lymphoid organs by various mechanisms^9–16^. Animal studies have shown high dose of PFOS treatment causes atrophy of the thymus and spleen^10, 15^. And *in vivo* PFOS treatment inhibits Th1 responses while Th2 responses are promoted^9, 13, 14^. Being a paper-packaging material and a contaminant in the water, PFOS can frequently be absorbed through the oral route and accumulate in the intestine, thus modulate intestinal immunity under physiological and pathological conditions. However, it is not known whether and how PFOS affects the intestinal immune cells, especially during pathological conditions such as intestinal bacterial infections.

Mouse *Citrobacter rodentium* infection has been widely used as a model for studying human intestinal infections, such as *enteropathogenic E. coli and enterohemorrhagic E. coli* infection^17–19^. Innate and adaptive immune cells are activated by antigens derived from *C. rodentium* and exhibit immune defensive function to clear the pathogen. Th17 cells, one subset of T helper cells, are characterized by the expression of master transcription factor RAR-related orphan receptor gamma t (RORγt) and are important for protective immunity against *C. rodentium* ^20–22^. Besides Th17 cells, group 3 innate lymphoid cells (ILC3s) are believed to be crucial for controlling the expansion of *C. rodentium* at early phase of infection before Th17 cell responses are primed^21, 23, 24^. Both Th17 cells and ILC3s secrete IL-17 and IL-22, which are key cytokines required for clearing *C. rodentium* by stimulating epithelial cells to secrete anti-microbial peptides or through recruitment of neutrophils^25–27^. Th17 cells and ILC3s share a lot of features including cytokine production and profiles of transcription factor expression^28, 29^. Besides RORγt, aryl hydrocarbon receptor (Ahr) is another well-established transcription factor expressed by both Th17 cells and ILC3s, and is known to be a key factor regulating the function of Th17 cells and ILC3s^24, 30–35^. Notably, dioxins from the environmental contaminants act as agonistic or antagonistic ligands for Ahr^36^. Interestingly, some of the perfluoroalkyl acids have been reported to be able to activate Ahr^37^, raising the possibility that PFOS may regulate Th17 cells and ILC3s through activating Ahr in the intestine.

In this study, we determined the effect of PFOS on mouse *C. rodentium* infection. We found PFOS prevented the growth of *C. rodentium* at early stage of infection by promoting IL-22 production from ILC3 in an Ahr-dependent manner. However, PFOS exposure caused persistent inflammation in the intestine accompanied by decreased mucin production from goblet cells and dysbiosis, which finally led to a failure to clear *C. rodentium* at late phase of infection. Our finding reveals that PFOS exposure leads to a detrimental consequence in intestinal bacterial infection.

## Results

### Perfluorooctane sulfonate (PFOS) exhibits differential roles at different stages of intestinal bacterial infection

**Figure 1:**
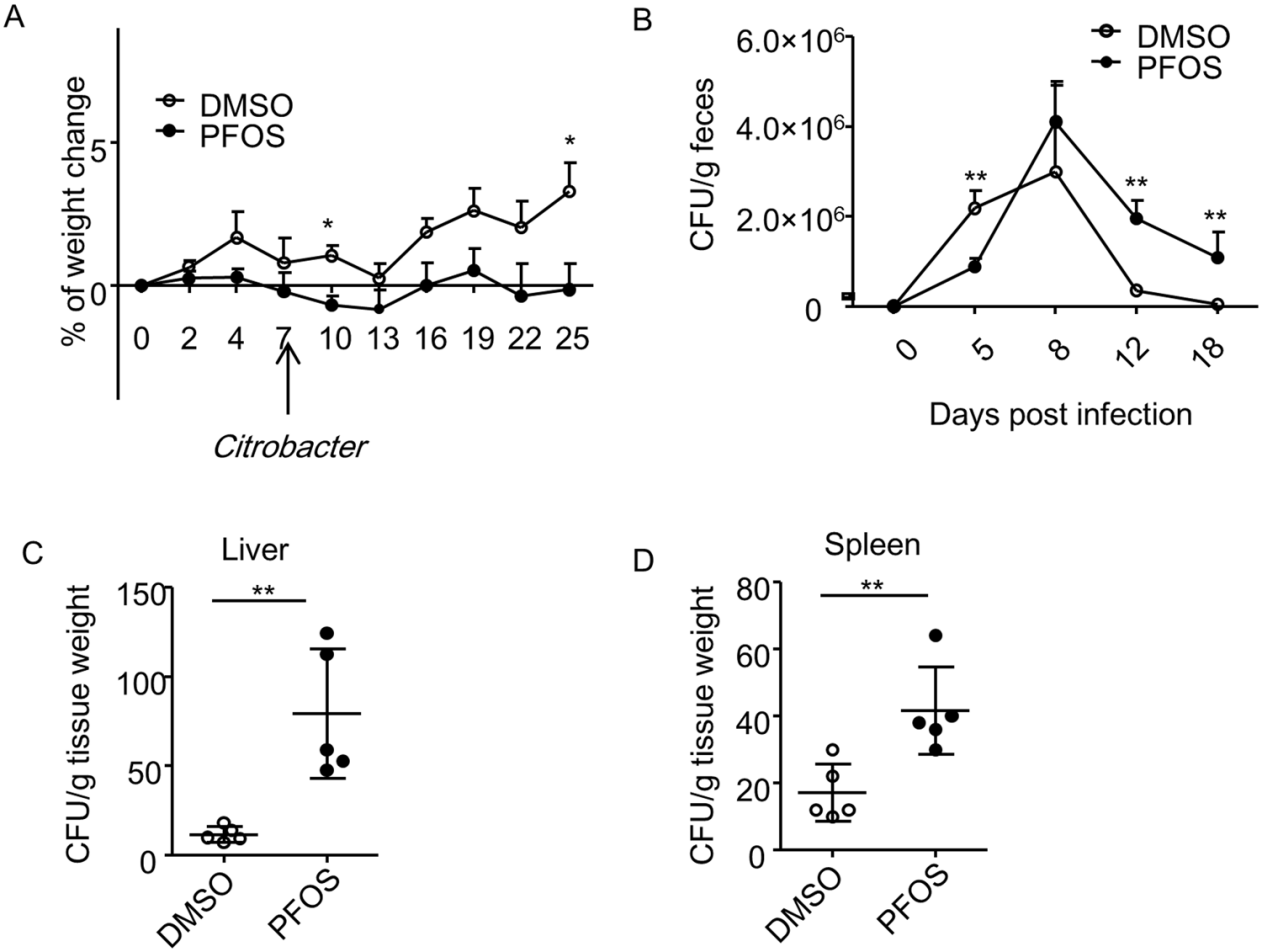
PFOS exhibits differential effects during different stages of mouse *C. rodentium* infection. (**A**) *Wild-type* mice were treated daily by oral gavage with DMSO or PFOS (2 mg/kg) in water for 7 days. Mice were then infected with 10^10^ Colony-forming unit (CFU) of *C. rodentium*. PFOS treatment continued on a daily basis through the whole time course of observation. Percentages of weight change of the two groups of mice before and after *C. rodentium* infection at indicated time points were shown. Data were pooled from 4 mice from each group. Error bars represent SEM. Data are representative of at least 3 independent experiments. (**B**) CFU counts of *C. rodentium* in the fecal pellets at indicated time points after infection were shown. Data were pooled from at least 4–8 mice from each group for all time points from two experiments. Error bars represent SEM. (**C**) CFU counts of *C. rodentium* from spleen and liver homogenates cultures at 21 days after infection were shown. Horizontal lines show the mean. Error bars represent SEM. The data are representative of three independent experiments.

To determine the effect of PFOS on intestinal infection, we infected mice with *Citrobacter rodentium* while treating mice with PFOS by oral gavage before and during the infection. We gavaged mice daily with PFOS at 2 mg/kg or vehicle control for 7 days before infecting mice with *C. rodentium*. Mice were continuously treated with PFOS during the whole course of observation. Though both PFOS treated and control group showed no obvious weight loss during *C. rodentium* infection, PFOS treated mice had less gain of weight after infection with *C. rodentium* compared to control, indicating potential sickness of PFOS treated mice (Fig. 1A). Under the steady state without *C. rodentium*, the difference in the change of body weight was not observed in PFOS treated mice compared to control, implying the pathogenic role of PFOS mainly exists in intestinal infection (Figure S1). On day 5 after *C. Rodentium* infection, we observed a significantly lower pathogen burden in PFOS treated mice compared to control group (Fig. 1B). This data suggests PFOS has a protective effect at early phase of *C. rodentium* infection. However, *C. rodentium* load in PFOS treated mice reached a comparable level to control group at day 8 after infection, which is considered to be the peak phase of this model (Fig. 1B)^38^. And on day 12 after infection, although both control and PFOS treated mice showed a sign for clearance of *C. rodentium*, PFOS gavaged mice manifested a much less extent of pathogen clearance compared to control group (Fig. 1B). The enhanced *C. rodentium* burden in PFOS treated mice compared to control group lasted till as late as day 18 post infection, suggesting a pathogenic role of PFOS at late phase of *C. rodentium* infection (Fig. 1B). The increased level of *C. rodentium* in PFOS treated mice was also observed in the liver and the spleen compared to control, although the absolute amount of bacteria burden was not high enough to cause lethality of any individual mouse (Fig. 1C and D). The above data suggest PFOS treatment limits the expansion of *C*. *rodentium* at early phase of the infection. However, it causes a failure to clear the pathogen efficiently at late stage of infection.

### PFOS promotes anti-microbial defense at early phase of *C. rodentium* infection by enhancing IL-22 production from ILC3 cells

**Figure 2:**
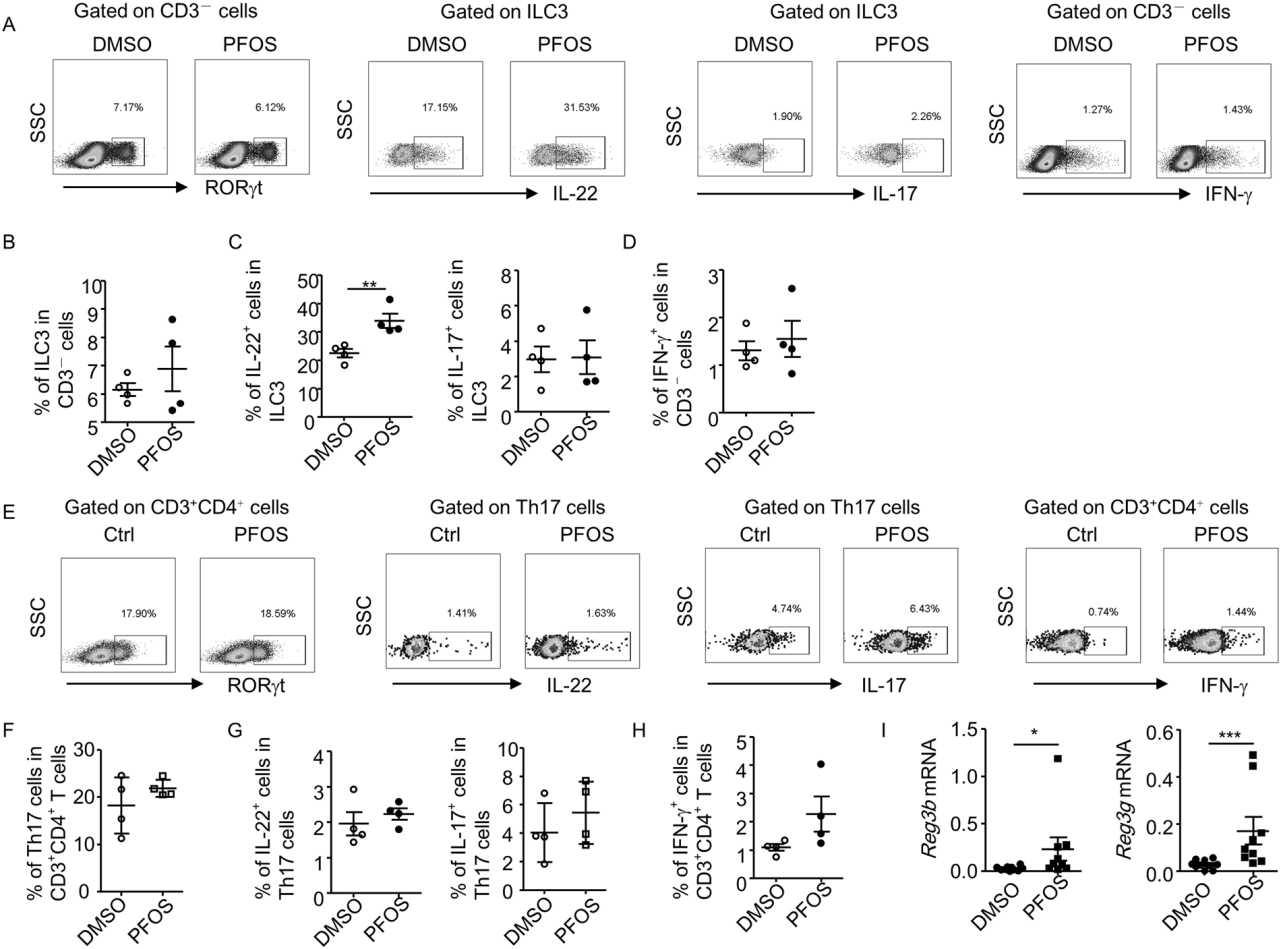
PFOS enhances IL-22 production from ILC3 at early phase of *C. rodentium* infection. On day 5 post *C. rodentium* infection, large intestinal lamina proprial lymphocytes were isolated from each group and analyzed by flow cytometry. Cells were stimulated with PMA and ionomycin for 4 hr before analysis. (**A** and **E**) The expression of CD3, CD4, RORγt, IL-17, IL-22 and IFN-γ was analyzed by flow cytometry. (**B**) Percentages of ILC3 (CD3^−^RORγt^+^ cells) gated on CD3^−^ cells were shown. (**C**) Percentages of IL-22 and IL-17 expression gated on ILC3 were shown. (**D**) Percentages of IFN-γ gated on CD3^−^ cells were shown. (**F**) Percentages of Th17 cells (CD3^+^CD4^+^RORγt^+^ cells) gated on CD3^+^CD4^+^ cells were shown. (**G**) Percentages of IL-22 and IL-17 production gated on Th17 cells were shown. (**H**) Percentages of IFN-γ gated on CD3^+^CD4^+^ cells were shown. Horizontal lines show the mean. Error bars represent SEM. Data are representative of two independent experiments. (**I**) mRNA expression of RegIIIβ and RegIIIγ in colon tissues was analyzed using realtime RT-PCR. Statistical analyses were performed using Mann-Whitney unpaired U test. Horizontal lines show the mean. Error bars represent SEM. Data are representative of two independent experiments.

ILC3s has been shown to play a key role in controlling *C. rodentium* infection, specifically at early phase of the infection, while Th17 cells exhibit immune defensive effect at late stage of infection^21^. Both ILC3s and Th17 cells produce IL-17 and IL-22, two major cytokines that are required for controlling intestinal pathogen infection^25–27^. Therefore, we analyzed the percentage of ILC3s and Th17 cells, as well as functional cytokine production from ILC3s and Th17 cells of large intestinal lamina proprial lymphocytes (LPLs) by flow cytometry. The percentage of ILC3s gated on CD3^−^ non-T cell population was similar between control and PFOS treated group (Fig. 2A and B). But the IL-22 production by ILC3 significantly enhanced in PFOS treated mice compared to control (Fig. 2A and C). Consistent with previous findings^24, 39^, ILC3 produced limited amount of IL-17, which was not affected by PFOS treatment (Fig. 2A and C). Percentage of IFN-γ production from CD3^−^ non-T cells in PFOS treated mice was comparable to control (Fig. 2D). Percentage of Th17 cells among all the CD4^+^ T cells was not changed in PFOS treated group compared to control mice (Fig. 2E and F). No obvious difference was observed in IL-17 or IL-22 production from Th17 cells in PFOS treated mice compared to control group (Fig. 2E and G). The production of IFN-γ by CD4^+^T cells in PFOS treated mice was on average but not significantly higher than control group, indicating a possibly pro-inflammatory role of PFOS in the intestine (Fig. 2E and H). We further evaluated the role on PFOS on ILC3 under the steady state without infection. Mice were treated with PFOS continuously for 11 days to match the same dose of PFOS at early phase of infection. No difference in the percentage of ILC3 was found in PFOS treated mice compared to control (Figure S2A). Interestingly, the production of IL-22 but not IL-17 from ILC3 was similarly enhanced by PFOS treatment without infection (Figure S2B and S2C). This suggests the promotion of IL-22 production by ILC3 was independent of infection. We further measured the expression of anti-microbial peptides, which are downstream of IL-22 and play essential role in clearance of *C. rodentium* during infection^25^. We consistently observed enhanced level of mRNA expression of RegIIIβ and RegIIIγ in colon tissues of PFOS treated mice (Fig. 2I). From above data, we reasoned the protective effect of PFOS at early stage of *C. rodentium* infection was mainly mediated by enhanced IL-22 production from ILC3.

### PFOS induces cytokine production by ILC3s and Th17 cells *in vitro*

**Figure 3:**
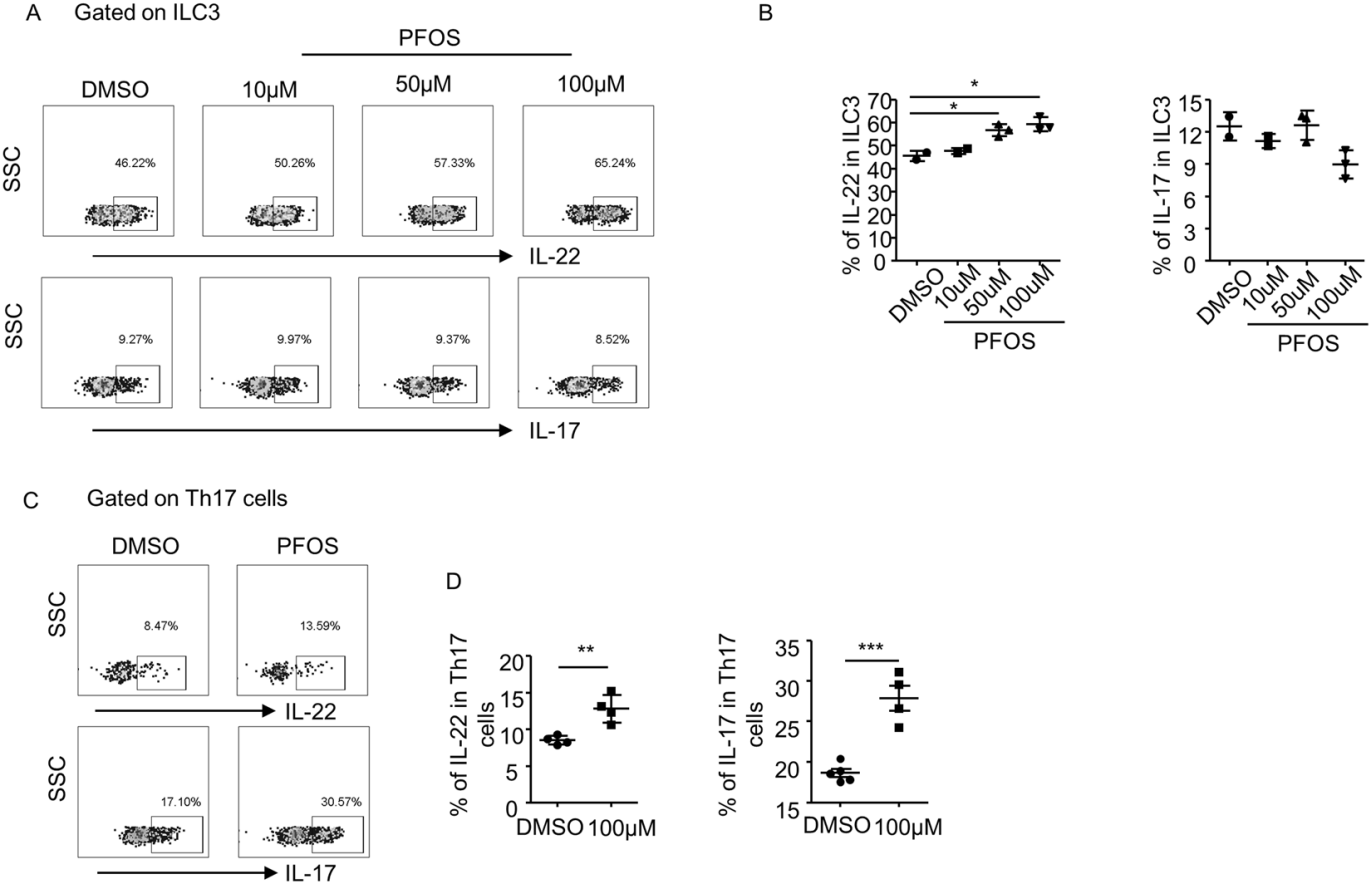
PFOS promotes cytokine production from intestinal ILC3 and Th17 cells *in vitro*. Large intestinal LPLs were isolated from wild-type mice and treated with indicated concentrations of PFOS or DMSO for 20 hr. Cells were stimulated with PMA and ionomycin for the last 4 h before harvested for flow cytometry analysis. (**A** and **C**) The expression of CD3, CD4, RORγt, IL-17 and IL-22 from indicated samples was analyzed by flow cytometry. (**B**) Percentages of IL-22 and IL-17 expression gated on ILC3 (CD3^−^RORγt^+^ cells) were shown. (**D**) Percentages of IL-22 and IL-17 expression gated on Th17 (CD3^+^CD4^+^RORγt^+^) cells were shown. Horizontal lines show the mean. Error bars represent SEM. Data are representative of two independent experiments.

IL-1β and IL-23 produced from antigen presenting cells are considered to be key drivers for IL-22 production from ILC3 during *C. rodentium* infection^25, 40, 41^. To determine whether PFOS promoted the function of ILC3s and Th17 cells by inducing IL-1β or IL-23, we isolated LPL from *C. rodentium* infected mice on day 5 post infection and analyzed the mRNA expression of IL-1β and IL-23 by real-time RT-PCR. We found no significant difference in IL-1β or IL-23 mRNA expression in the LPL of PFOS treated mice compared to control group (Figure S3A), suggesting these two cytokines were less likely to be involved in promoting IL-22 production from ILC3 by PFOS. We then isolated LPL cells from *wild-type* mice under the steady state and treated the cells with different doses of PFOS *in vitro*. We found PFOS potently promoted the production of IL-22 by ILC3 cells at the concentration of 50 uM and 100 uM after culturing for 20 hr, without cytotoxic effect (Fig. 3A,B and Figure S4). Consistently, IL-17 production by ILC3 was not affected by PFOS treatment *in vitro* (Fig. 3A and B). Interestingly, both IL-17 and IL-22 production from Th17 cells were enhanced by PFOS treatment at 100 uM (Fig. 3C and D). Moreover, no difference in IL-23 and IL-1β mRNA was affected by PFOS treatment *in vitro* (Figure S3B). Thus, PFOS was more likely to promote the function of ILC3 and Th17 cell through a cell-intrinsic mode.

### PFOS promotes the function of ILC3s and Th17 cells in an Ahr-dependent manner

**Figure 4:**
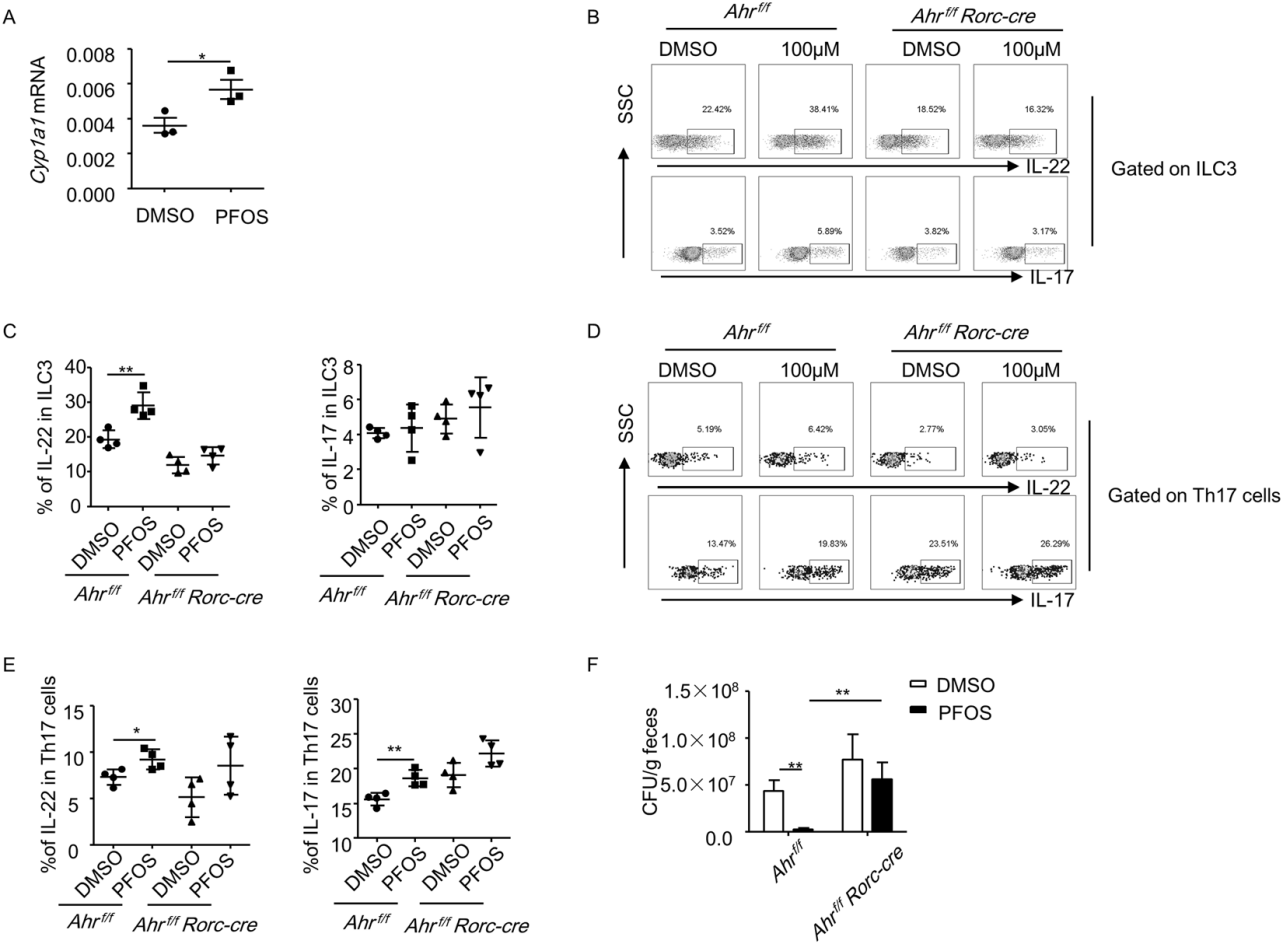
PFOS inhibites the growth of *C. rodentium* by promoting IL-22 production from ILC3 through an Ahr-dependent manner. (**A**) Large intestinal LPLs were isolated from *wild-type* mice and treated with PFOS at 100 uM or DMSO for 20 hr. mRNA expression of *Cyp1a1* in cultured LPLs was analyzed by real-time RT-PCR. (**B** to **E**) Large intestinal LPLs were isolated from *Ahr* ^*f/f*^ *RORc-cre* or *Ahr* ^*f/f*^ mice and treated with PFOS at 100 uM or DMSO for 20 hr. Cells were stimulated with PMA and ionomycin for the last 4 hr before harvested for flow cytometry analysis. (**B** and **D**) The expression of CD3, CD4, RORγt, IL-17 and IL-22 from indicated samples was analyzed by flow cytometry. (**C**) Percentages of IL-22 and IL-17 expression gated on ILC3 (CD3^−^RORγt^+^ cells) were shown. (**E**) Percentages of IL-22 and IL-17 expression gated on Th17 (CD3^+^CD4^+^RORγt^+^) cells were shown. (**F**) *Ahr* ^*f/f*^ *RORc-cre* or *Ahr* ^*f/f*^ mice were treated with PFOS at 2 mg/kg for 7 days before mice were infected with 10^10^ CFU of *C. rodentium*. CFU counts of *C. rodentium* in the fecal pellets from mice of indicated genotypes at day 5 post-infection were shown. Horizontal lines show the mean. Error bars represent SEM. Data are representative of two independent experiments.

Previous study suggests perfluoroalkyl acids act as an agonist for aryl hydrocarbon receptor (Ahr)^37^, a nuclear transcriptional factor which is highly expressed by various immune cells and crucial for the development and function of ILC3s and Th17 cells^24, 30–35^. Indeed, the mRNA expression of Cyp1a1, a direct target gene of Ahr, was significantly elevated in the LPL by PFOS treatment (Fig. 4A). To further determine whether PFOS promoted the function of Th17 cells and ILC3s through Ahr in a cell-intrinsic mechanism, we crossed *Ahr* ^*f/f*^ mouse to *RORc-cre* mouse to delete Ahr specifically in ILC3 and T cells (*Ahr* ^*f/f*^ *RORc-cre*). We then treated LPL isolated from either *Ahr* ^*f/f*^ *RORc-cre* or *Ahr* ^*f/f*^ mice with PFOS *in vitro*. While PFOS potently enhanced the IL-22 production by ILC3 from LPL of *Ahr* ^*f/f*^ mice, this effect was ablated in *Ahr* ^*f/f*^ *RORc-cre* mice (Fig. 4B and C). IL-22 production from ILC3 on a per-cell-based level was also enhanced by PFOS in ILC3 from *Ahr* ^*f/f*^ but not *Ahr* ^*f/f*^ *RORc-cre* mice indicated by the mean fluorescence intensity (MFI) of IL-22 gated on ILC3s (Figure S5). Similarly, IL-22 and IL-17 production from Th17 cells was enhanced in *Ahr* ^*f/f*^ mice but not in *Ahr* ^*f/f*^ *RORc-cre* mice by PFOS treatment (Fig. 4D and E). IL-17 production from ILC3 was not obviously changed by PFOS treatment in both *Ahr* ^*f/f*^ *RORc-cre* mice and *Ahr* ^*f/f*^ mice (Fig. 4B and C). Thus, we conclude PFOS promotes the function of ILC3 and Th17 *in vitro* through Ahr.

We then investigated whether PFOS protected mice from *C. rodentium* infection through activating Ahr *in vivo*. We found while PFOS efficiently inhibited the expansion of *C. rodentium* in *Ahr* ^*f/f*^ mice on day 5 post infection, *C. rodentium* count was significantly higher in *Ahr* ^*f/f*^ *RORc-cre* mice and PFOS failed to inhibit the growth of the pathogen (Fig. 4F). This data suggests Ahr expression on ILC3s and T cells is required for the protective effect of PFOS at early phase of *C. rodentium* infection. Since Th17 cell immunity was not affected by PFOS treatment during early phase of *C. rodentium* infection, PFOS mainly activated Ahr in ILC3 to promote the production IL-22 by ILC3, which is essential for controlling the expansion of *C. rodentium*.

### Pro-inflammatory effect of PFOS at late stage of *C. rodentium* infection

**Figure 5:**
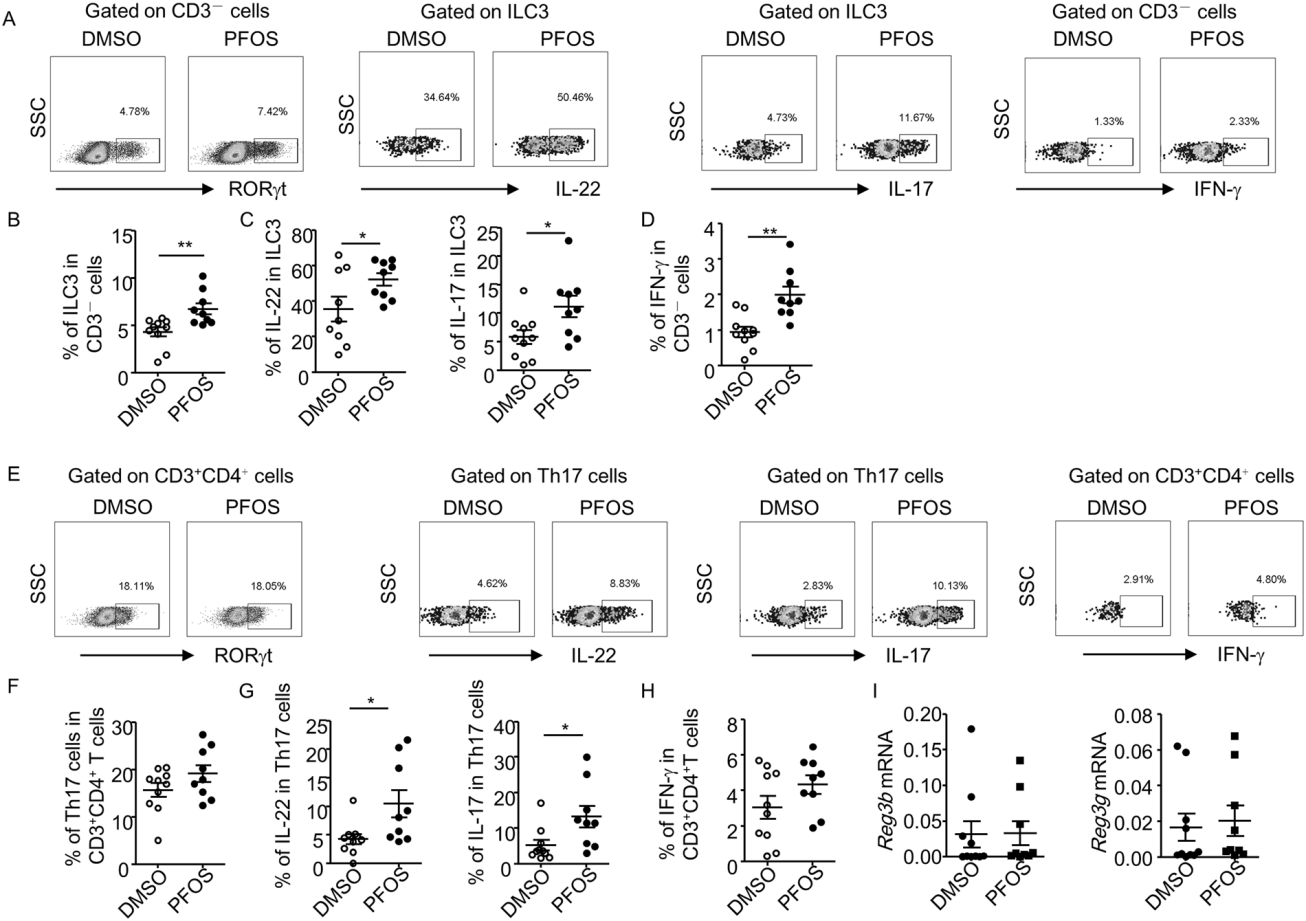
PFOS promotes both innate and adaptive production of IL-17 and IL-22 at late phase of *C. rodentium* infection. On day 12 post *C. rodentium* infection, large intestinal lamina proprial lymphocytes were isolated from each group and analyzed by flow cytometry. Cells were stimulated with PMA and ionomycin for 4 hr before analysis. (**A** and **E**) The expression of CD3, CD4, RORγt, IL-17, IL-22 and IFN-γ was analyzed by flow cytometry. (**B**) Percentages of ILC3 (CD3^−^RORγt^+^ cells) gated on CD3^−^ cells were shown. (**C**) Percentages of IL-22 and IL-17 expression gated on ILC3 were shown. (**D**) Percentages of IFN-γ gated on CD3^−^ cells were shown. (**F**) Percentages of Th17 cells (CD3^+^CD4^+^RORγt^+^ cells) gated on CD3^+^CD4^+^ cells were shown. (**G**) Percentages of IL-22 and IL-17 production gated on Th17 cells were shown. (**H**) Percentages of IFN-γ gated on CD3^+^CD4^+^ cells were shown. Horizontal lines show the mean. Error bars represent SEM. Data were pooled from three independent experiments. (**I**) mRNA expression of RegIIIβ and RegIIIγ in colon tissues was analyzed using realtime RT-PCR. Statistical analyses were performed using Mann-Whitney unpaired U test. Horizontal lines show the mean. Error bars represent SEM. Data are representative of two independent experiments.

Although PFOS controlled the growth of *C. rodentium* at early phase of infection, the pathogen greatly expanded at peak phase (day 8 after infection) and PFOS treated mice failed to clear *C. rodentium* efficiently after day 12 post infection (Fig. 1B). We examined whether the defective defense against *C. rodentium* in PFOS treated mice was caused by loss of function of ILC3s and Th17 cells at late stage of the infection. On day 12 post infection, we found the percentage of ILC3s gated on CD3^−^ non-T cell population increased in PFOS treated group compared to control mice (Fig. 5A and B). Moreover, the production of IL-17 and IL-22 by ILC3 was significantly higher than control mice (Fig. 5A and C). Notably, IFN-γ production from CD3^−^ non-T cells also increased in PFOS treated mice compared to control, indicating a pro-inflammatory role of PFOS during *C. rodentium* infection (Fig. 5A and D). Th17 cell response on day 12 of infection was much higher than early phase (day 5) (Figs 2E and 5E). No difference in the percentage of Th17 cells gated on CD4^+^T cells was changed by PFOS treatment (Fig. 5E and F). However, the level of IL-17 and IL-22 produced by Th17 significantly enhanced in PFOS treated mice compared to control group (Fig. 5E and G). IFN-γ production by CD4^+^ T cells was on average but not significantly higher in PFOS treated mice than control at late stage of infection (Fig. 5E and H). Notably, PFOS consistently enhanced IFN-γ production from both CD3^−^ non-T cells and CD4^+^ T cells *in vitro*, further indicating the pro-inflammatory role of PFOS in intestinal immunity (Figure S6A–S6D). The above data indicate IL-17 and IL-22 from both the innate and adaptive sources are boosted by PFOS treatment, likely due to a further accumulation of the compound locally. Nevertheless, the expression of RegIIIβ and RegIIIγ, which are downstream of IL-22, was not higher in PFOS treated mice compared to control (Fig. 5I). And PFOS treated mice failed to clear the pathogen efficiently despite an enhanced defensive immune responses during late phase of *C. rodentium* infection.

We also evaluated the role of long-term PFOS treatment on immune subsets and cytokine production without *C. rodentium* infection. We treated mice for 17 consecutive days to match the dose of PFOS at late phase of infection. Compared to the infection status where an extensive pro-inflammatory cytokine production from both ILC3 and Th17 cells was induced in PFOS treated mice (Fig. 5), only the induction of IL-22 from ILC3 and Th17 cells was consistently found in PFOS treated group under the steady state (Figure S7A, S7C, S7E and S7G). There was no difference in percentages of ILC3 or Th17 cells in PFOS treated mice compared to control (Figure S7A, S7B, S7E and S7F). The levels of IL-17 and IFN-γ production from both innate cells and T cells were comparable between two groups (Figure S7). The above data indicate the induction of IL-22 by PFOS *in vivo* is independent of infection. However, the pro-inflammatory effect of PFOS to enhance IL-17 and IFN-γ production only occurs during infectious status.

### Persistent PFOS exposure results in reduced mucin production and dysbiosis

**Figure 6:**
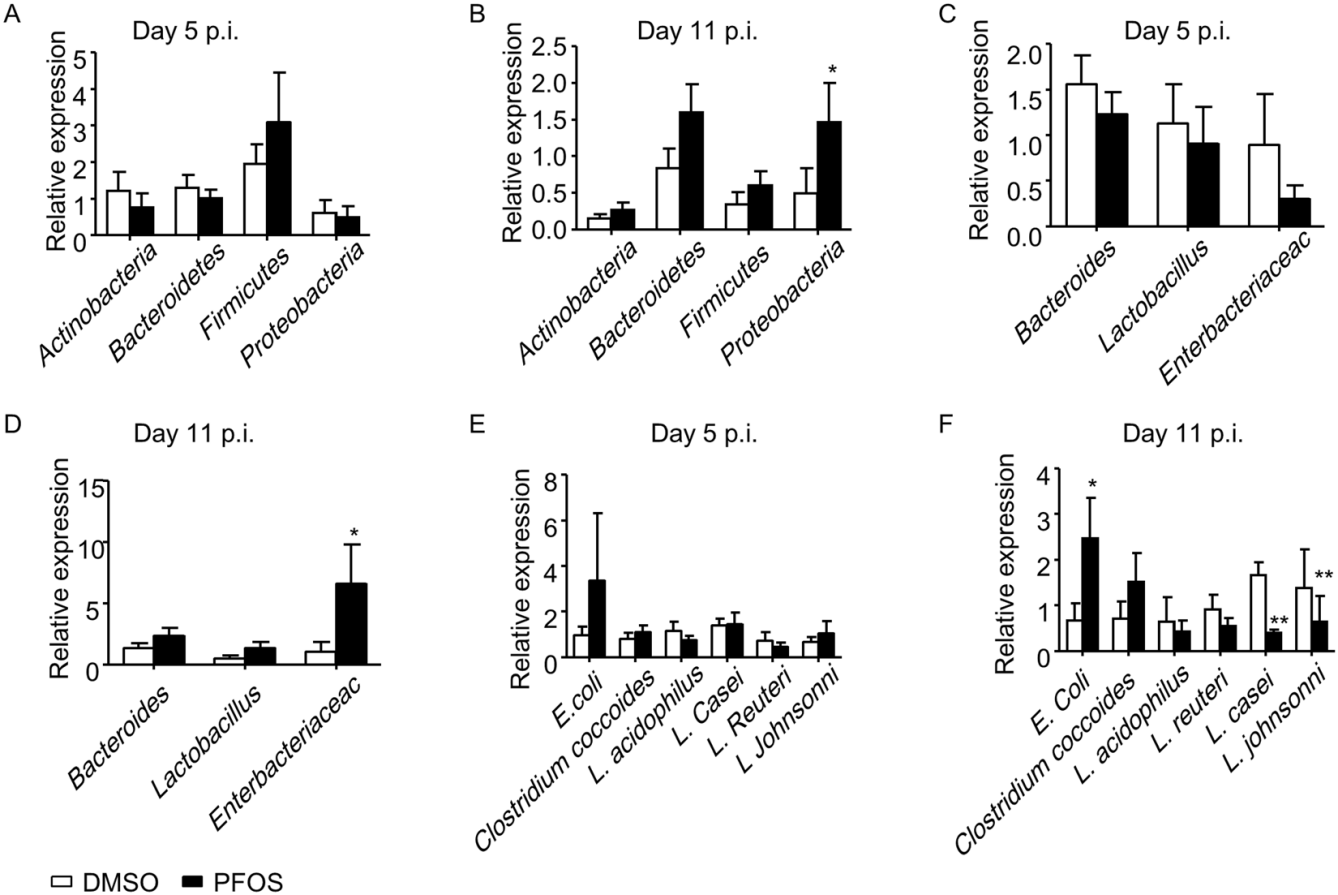
PFOS treatment results in dysbiosis at late phase of *C. rodentium* infection. *Wild-type* mice were treated with PFOS and infected with *C. rodentium* as described in Fig. 1. (**A**,**C** and **E**) The expression of indicated commensals in the feces of PFOS treated mice and control at day 5 post *C. rodentium* infection (p.i.) was analyzed by real-time PCR. (**B**,**D** and **F**) The expression of indicated commensals in the feces of PFOS treated mice and control at day 11 post *C. rodentium* infection (p.i.) was analyzed by real-time PCR. Statistical analyses were performed using Mann-Whitney unpaired U test. Error bars represent SEM. Data were pooled from three independent experiments.

The paradoxical phenotype of enhanced ILC3 and Th17 responses and increased bacterial burden in PFOS treated mice made us to think other mechanisms should be accounted for the failure to clear *C. rodentium*. Previous reports have suggested the colonization of *C. rodentium* is associated with distribution of commensal flora in the gut^42–45^. And dysbiosis may cause increase of susceptibility to *C. rodentium* infection^46^. Therefore, we checked the expression of different bacterial commensals in the feces of PFOS treated mice and control mice at both early and late phase during *C. rodentium* infection using real-time PCR. We first compared the expression of major bacterial phylum in PFOS treated mice and control mice during infection. No difference was found in the expression of *Actinobacteria*, *Bacteroidetes* and *Firmicutes* in PFOS treated mice and control mice at both early and late phase of *C. rodentium* infection (Fig. 6A and B). However, the expression of *Proteobacteria* was significantly higher in PFOS treated mice at the late but not early stage of *C. rodentium* infection (Fig. 6B). We further analyzed the expression of commensals at the genus or family level which belong to the *Bateroidetes*, *Firmicutes* and *Proteobacteria* phylum. No difference was found in all the analyzed commensals at the early stage of infection between PFOS treated group and control (Fig. 6C). Nevertheless, *Enterobacteriaceae* was found to be significantly higher in PFOS treated mice than control mice at the late stage of infection (Fig. 6D). The increase of *Enterobacteriaceae* and *Proteobacteria* was likely due to expansion of *C. rodentium* in PFOS treated mice since *C. rodentium* belongs to the *Proteobacteria* phylum and *Enterobacteriaceae* family^47^. We thus analyzed the expression of commensals at the species level (Fig. 6E and F). Interestingly, *E. coli* was found to increase in PFOS treated group compared to control group at late but not early phase of infection, suggesting the presence of dysbiosis specifically at late phase of infection (Fig. 6F). We further analyzed 4 types of bacterial species in the *Lactobacillus* genus, which has been shown to play regulatory roles during intestinal inflammation^42–45^. We found *Lactobacillus johnsonni* and *Lactobacillus casei* decreased in PFOS treated mice at late but not early phase of infection, while *Lactobacillus acidophilus* and *Lactobacillus reuteri* was not affected by PFOS at both early and late phase of the infection (Fig. 6E and F). Given the reported protective effect of *Lactobacillus johnsonni* and *Lactobacillus casei* in *C. rodentium* infection and epithelial cell functions^48, 49^, dysbiosis caused by PFOS treatment at late stage of *C. rodentium* infection could contribute to the failure of the host to clear *C. rodentium* efficiently.

**Figure 7:**
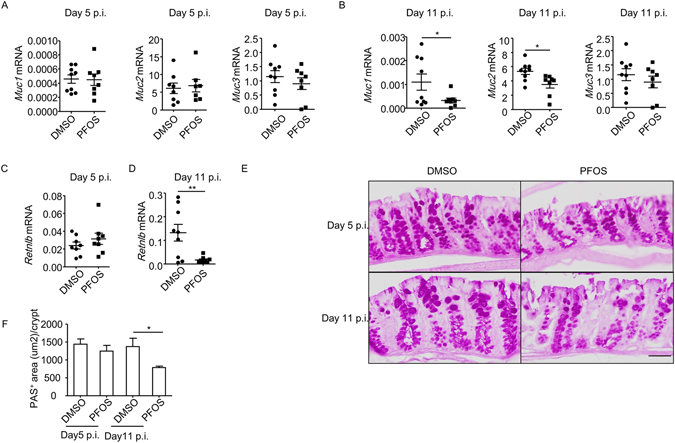
PFOS ablates function of goblet cells at late phase of *C. rodentium* infection. *Wild-type* mice were treated with PFOS and infected with *C. rodentium* as described in Fig. 1. (**A**) mRNA expression of Muc1, Muc2 and Muc3 at day 5 post *C. rodentium* infection in colon tissues was analyzed by realtime RT-PCR. (**B**) mRNA expression of Muc1, Muc2 and Muc3 at day 11 post *C. rodentium* infection in colon tissues was analyzed by realtime RT-PCR. (**C**) mRNA expression of *Retnlb* at day 5 post *C. rodentium* infection in colon tissues was analyzed by realtime RT-PCR. (**D**) mRNA expression of *Retnlb* at day 11 post *C. rodentium* infection in colon tissues was analyzed by realtime RT-PCR. (**A**–**D**) Horizontal lines show the mean. Error bars represent SEM. Data were pooled from two independent experiments. (**E**) Paraffin-embedded colon sections were stained with periodic acid-schiff (PAS). Magnification is 20 × (Scale bar, 50 um). (**F**) PAS^+^ areas per crypt were quantified by ImageJ Software. Data were pooled from three mice from indicated group. Error bars represent SEM. Data are representative of two independent experiments.

Mucins produced by goblet cells in the intestinal epithelium are crucial for maintaining a healthy community of microbiota in the gut^50, 51^. Pro-inflammatory cytokines including IL-17 and IFN-γ have been indicated to cause tissue damage or result in loss of goblet cells during intestinal inflammation^52, 53^. We suspect the enhanced inflammation induced by PFOS at late stage of *C. Rodenium* infection may cause a defective mucin production by goblet cells. We then analyzed the mRNA expression of mucins in colon tissues by real-time RT-PCR. We observed significant reduction of mRNA expression of mucin 1 and mucin 2 at late stage but not early stage of *C. rodentium* infection in PFOS treated mice compared to control (Fig. 7A and B), while expression of mucin 3 was comparable between two groups (Fig. 7A and B). Interestingly, we found the mRNA expression of RELM-β, a resistin-like molecule specifically expressed by goblet cells, decreased in PFOS treated mice at late but not early phase of *C. rodentium* infection (Fig. 7C and D). The combinatorial downregulation of mucins and RELM-β may result in dysbiosis featured by increased *E. coli* and decreased *Lactobacillus* species^51^.

We also measured the level of mucins and RELM- β under the steady state without infection. Short-term PFOS treatment didn’t affect expression of mucins (Figure S8A). Intriguingly, REML-β was found to be enhanced by PFOS treatment (Figure S8C). Furthermore, a reduction of mucin 2 was found in mice with long-term PFOS treatment compared to control (Figure S8B). However, no difference was observed in mucin 1, 3 or RELM- β expression with long-term PFOS treatment (Figure S8B and S8D). The above data suggest long-term treatment of PFOS suppresses the expression of mucin 2 independent of infection. Nevertheless, infection resulted in a more profound dysfunction of epithelial cells, indicated by decreased level of mucin 1 and RELM- β at the late phase (Fig. 7B and D). This was likely caused by a more extensive inflammation triggered by PFOS during infection, which finally led to dysbiosis.

We further examined the mucin production from intestinal epithelial cells using periodic acid-schiff (PAS) staining during infection. Consistently, PFOS treated mice manifested reduced PAS^+^ area per crypt, indicating reduced mucin or number of goblet cells, compared to control group at late but not early stage of *C. rodentium* infection (Fig. 7E and F). From above data, we conclude that PFOS causes decreased mucin production from intestinal epithelial cells, which may lead to dysbiosis and exacerbation of *C. rodentium* infection at late phase.

## Discussion

Perfluorooctane sulfonate (PFOS) has been manufactured to be used as surfactants and repellents in the industry for its desirable properties of high surface activity and water and oil repellence. Due to its long half-life for serum clearance and resistance to environmental degradation, PFOS is bio-accumulative and remains to be a threat to human health^3, 4, 8, 54, 55^. Being widely used in food-packaging material and non-stick pans, PFOS absorption through the oral route is considerably common. Thus, the accumulation of PFOS in the intestine may have deleterious effect on intestinal diseases, such as intestinal infection, intestinal autoimmune diseases and tumor. In this study, we determined the effect of PFOS on intestinal immunity and intestinal infection using a mouse model of *Citrobacter rodentium* infection, which recapitulates human *enteropathogenic E. coli and enterohemorrhagic E. coli* infection^17–19^. We have found PFOS affects the outcome of *C. rodentium* infection through modulating intestinal immunity and microbiota. At early stage of infection, PFOS prevents the expansion of *C. rodentium* by promoting the IL-22 production from the group 3 innate lymphoid cells (ILC3s) through activating aryl hydrocarbon receptor (Ahr), which is a key transcription factor known to regulate the development and function of ILC3s. However, consistent exposure to PFOS finally leads to a failure to clear the *C. rodentium* at late stage, mainly due to dysbiosis accompanied by persistent inflammation and reduced mucin production by goblet cells. Our study brought out the caution that the pro-inflammatory effect of PFOS in the intestine may result in dysbiosis and failure of clearing intestinal pathogen.

In our study, we have found PFOS promoted the cytokine production from both ILC3s and Th17 cells, which are two major immune cell subsets that are required for defense against *C. rodentium* ^21, 23–27^. At the primary expansion phase of *C. rodentium* infection (day 5), PFOS boosted the production of IL-22 from ILC3 without having any effects on Th17 cells. At late stage of infection, PFOS exhibited more pro-inflammatory effect indicated by increased IL-17, IL-22 and IFN-γ by innate cells and enhanced Th17 cell immunity. The delayed effect of PFOS on Th17 cells *in vivo* maybe because Th17 cells are less sensitive to PFOS mediated effect than ILC3s. Thus, a longer time of exposure and accumulation of PFOS is required to boost Th17 cell responses. Indeed, *in vitro* stimulation of ILC3 to secrete IL-22 required a much lower concentration of PFOS than Th17 cells, indicating ILC3 is more sensitive than Th17 to PFOS treatment.

Given the immune defensive effect of IL-17, IL-22 and IFN-γ, the increased level of the above inflammatory cytokines appeared contradictory to the failure to clear *C. rodentium* at late phase of infection in PFOS treated mice. However, the persistent inflammation caused by accumulative PFOS during the infection may be overt, thus result in tissue damage and loss of goblet cells, which may finally lead to dysbiosis and failure to clear *C. rodentium*. Notably, despite enhanced IL-22 level at both early and late stage of infection in PFOS treated mice, the expression of anti-microbial peptides was higher only at early phase of infection. Since anti-microbial peptides are well-known as targets of IL-22^25^, the failed induction of anti-microbial peptides in response to higher IL-22 in PFOS treated mice at late phase of infection may due to a damage of epithelial cells caused by overt inflammation. Previous studies have revealed the pathogenic role of IL-17 and IFN-γ in tissue damage and loss of goblet cells^52, 53^. We found IFN-γ production from CD3^−^non-T cells significantly increased at late phase of *C. rodentium* infection. A trend of increased IFN-γ production by CD4^+^ T cells was also observed at both early and late phase of *C. rodentium* infection. And IL-17 production from both ILC3 and Th17 cells was upregulated during late phase of *C. rodentium* infection. In addition, we found the innate and T cell production of IFN-γ was consistently enhanced by PFOS *in vitro*, suggesting a promotive effect of PFOS on type 1 immunity. The molecular mechanism of how PFOS promoted type 1 immunity in the intestine remains to be determined.

The failure to clear *C. rodentium* in PFOS treated mice at late phase of infection was accompanied by dysbosis featured by enhanced level of *E. coli* and decreased level of *Latobacillus johnsonni* and *Lactobacillus casei*. Though the causal link between *E. coli* expansion and *C. rodentium* growth is unclear, the protective effect of *Latobacillus johnsonni* and *Lactobacillus casei* in *C. rodentium* infection and epithelial barrier function has been shown in previous studies^48, 49^. Since mucins produced by goblet cells have been shown to be essential for the maintenance of microbial homeostasis and defense against *C. rodentium* in the gut^50, 51^, we reasoned goblet cell loss and dysbiosis at late phase of infection contributes to the failure of clearing *C. rodentium* by PFOS treated mice. Indeed, histology analysis revealed decreased mucin production by goblet cells in PFOS treated mice at late but not early phase of *C. rodentium* infection, which was commensurate with no observed dysbiosis in PFOS treated group at early phase of infection. And mRNA production of mucin 1 and mucin 2 was significantly lower in PFOS treated mice. In addition, RELM-β, another protein specifically produced by goblet cells and required for prevention of dysbiosis in synergy with mucin 2, also decreased in PFOS treated mice at late stage of infection^51^. Loss of goblet cells in PFOS treated mice may be caused by the persistence of pro-inflammatory responses, including increased IFN-γ, which may also be the reason for reduced RELM-β^52, 56^.

The effect of PFOS on ILC3 is organ-specific because ILC3s and Th17 cells are specifically abundant in the intestine but rare in other organs under the steady state in mice^57–59^. PFOS has been reported to affect the immune cells in the central and peripheral lymphoid organs by various mechanisms^9–12, 14–16^. High dose of PFOS exposure has been shown to cause atrophy of the thymus and spleen, as well as the percentages of T cell subsets in the spleen^10, 15^. In this study, we used a previously reported low dose of PFOS to avoid direct toxicity to the thymus and spleen^10^. Except for the role of PFOS in promoting Th17 cell responses in the gut, similar effect is likely to occur in different autoimmune disorders where Th17 cells are pathogenic, such as multiple sclerosis and rheumatoid arthritis^60, 61^. Thus, it brings out an alert that PFOS accumulation may be detrimental for autoimmune diseases. Epidemiologic and experimental studies are called for further evaluation for the correlation of PFOS accumulation and autoimmune diseases.

## Methods

### Mice

*Wild-type* mice were purchased from Shanghai SLAC Laboratory Animal Co. *Ahr* ^*f/f*^ and *RORc-cre* mice were purchased from Jackson Laboratory. *Ahr* ^*f/f*^ *RORc-cre* mice were generated by crossing *Ahr-*floxed mice^62^ with *RORc-cre* mice^63^. All mice used in this study are on C57BL/6 background and maintained in specific pathogen-free conditions. All mice used in this study were littermate controlled, gender-matched and were 6–8 weeks old. All animal experiments were performed in compliance with the guide for the care and use of laboratory animals and were approved by the institutional biomedical research ethics committee of the Shanghai Institutes for Biological Sciences, Chinese Academy of Sciences.

### Chemicals

PFOS (heptadecafluorooctanesulfonic acid potassium salt, CAS 2795–39–3, purity >98%) was purchased from Sigma-Aldrich, dissolved in DMSO (100 mM), and stored at −20 °C as stock solution for *in vivo* and *in vitro* experiments. For *in vivo* treatments, PFOS (2 mg/kg) or same volume of DMSO was dissolved in water containing 0.5% Tween 20 and mice were gavaged 200 ul per day.

### *C. rodentium* Infection and Colony-Forming Unit (CFU) Counts

*C. rodentium* strain DBS100 (ATCC 51459; American Type Culture Collection) was cultured overnight and bacterial concentration was calculated by measuring optical density at a wavelength of 600 nm (OD600) with spectrometer. Mice were treated by oral gavage with 10^10^ CFU *C. rodentium* in 200 ul PBS. Body weight was measured. Fecal pellets, liver and spleen were collected, weighed and then homogenized in sterile PBS. Serially diluted homogenates were plated on MacConkey agar plates. *C. rodentium* colonies were identified based on morphology after 24 hr of incubation at 37 °C.

### Isolation of Large Intestinal Lamina Proprial Lymphocytes (LPLs)

The isolation of large intestinal lamina proprial lymphocytes was done as previously described^24^. Briefly, large intestines were dissected. Fat tissues were removed. Intestines were cut open longitudinally and washed in PBS. Intestines were then cut into 3 pieces, washed and shaken in PBS containing 1 mM DTT for 10 min at RT. Intestines were incubated with shaking in PBS containing 30 mM EDTA and 10 mM HEPES at 37 °C for 10 min for two cycles. The tissues were then digested in RPMI1640 medium (Invitrogen) containing DNase I (Sigma) (150 ug/ml) and collagenase VIII (Sigma) (150 U/ml) at 37 °C in 5% CO_2_ incubator for 1.5 hr. The digested tissues were homogenized by vigorous shaking and passed through 100 um cell strainer. Mononuclear cells were then harvested from the interphase of an 80% and 40% Percoll gradient after a spin at 2500 rpm for 20 min at room temperature.

### Flow Cytometry and Antibodies

Anti-mouse CD16/32 antibody was used to block the non-specific binding to Fc receptors before all surface stainings. All antibodies used for flow cytometry were purchased from eBioscience except for α-IL-22, which was purified from a hybridoma cell line (ATCC, PTA-7319) and labeled with biotin with a EZ-Link Micro Sulfo-NHS-LC-Biotinylation Kit (Pierce). For nuclear stainings, cells were fixed and permeabilized using a Mouse Regulatory T Cell Staining Kit (eBioscience). For cytokine stainings, cells were stimulated by PMA (Sigma) (50 ng/ml) and ionomycin (Sigma) (500 ng/ml) for 2 hr, and then treated with Brefeldin A (Sigma) (2 µg/ml) for another 2 hr before cells were harvested for analysis. Dead cells were stained with Live and Dead violet viability kit (Invitrogen) and were gated out in analysis. Flow cytometry data were collected using the Gallios flow cytometer (Beckman) and analyzed by FlowJo software (Tree Star Inc.)

### Cell culture

Purified large intestinal lamina proprial lymphocytes were cultured with DMSO or PFOS for 20 hr in IMDM medium (Hyclone), supplemented with 10% FBS (Gibco), 2 mM L-Glutamine (Gibco) and 100 U/ml Penicillin (Gibco), 100 ug/ml Streptomycin (Gibco) at 37 °C with 5% CO_2_. The concentration of DMSO in both control and PFOS group was 0.1%.

### Detection of mRNA by Real-time RT-PCR

RNA from control or PFOS treated large intestinal LPLs was isolated with Trizol reagent (Invitrogen). cDNA was synthesized using GoScript™ Reverse Transcription kit (Promega). Real-time PCR was performed using SYBR Green (Bio-rad). Reactions were run with the Mx 3000 P Q-PCR System (Angilent). The results were displayed as relative expression values normalized to β-actin. Primers used in this study were shown in Table S1.

### Bacterial DNA extraction and Real-time PCR

Fecal pellets were collected and total bacterial DNA was extracted using the Stool DNA Kit (Omega Biotek). Quantitative PCR for the 16 S rRNA gene was performed with SYBR Green (Bio-Rad) and normalized to total bacterial DNA. Reactions were run with the Mx 3000 P Q-PCR System (Angilent). Primers used in this study were shown in Table S2.

### Histological Analysis

The colonic swiss-roll was fixed with 4% formaldehyde and embedded in paraffin. Tissues were cut into 7 μm sections for periodic acid-schiff (PAS) staining to evaluate mucin production from epithelial cells. Sections were examined by Olympus light microscope (vs120). ImageJ software (National Institutes of Health, Bethesda, MD USA) was used to quantify area of PAS positive cells. PAS^+^ area in four fields of around 2.25 mm^2^ from each section was quantified and divided by number of crypts to obtain area of PAS^+^ cells per crypt.

### Statistical methods

Unless otherwise noted, statistical analyses were performed with the unpaired Student’s t test on individual biological samples. Linear regression analysis was performed with GraphPad Prism. ∗p < 0.05; ∗∗p < 0.01; ∗∗∗p < 0.001.

## Electronic supplementary material

Supplementary information
