# Supplementary material for: Perfluorooctane sulfonate affects intestinal immunity against bacterial infection
Source: Sci Rep. 2017 Jul 12;7:5166. doi: 10.1038/s41598-017-04091-z (PMC5507904; doi:10.1038/s41598-017-04091-z)
Supplement: Supplementary file 1 — Supplementary information [file 41598_2017_4091_MOESM1_ESM.pdf]

Supplemental Information

Perfluorooctane sulfonate affects intestinal immunity against bacterial infection

Caixia Suo, Zhiqin Fan, Liang Zhou and Ju Qiu

Inventory of Supporting Online Materials

- Figure S1 PFOS has no effect on change of body weight at steady state
- Figure S2 Short-term treatment of PFOS promotes the IL-22 production from ILC3 under the steady state
- Figure S3 PFOS has no impact on IL-1β and IL-23 mRNA expression
- Figure S4 PFOS has no cytotoxic effect *in vitro*
- Figure S5 PFOS promotes IL-22 production from ILC3 on a per-cell-based level *in vitro* through Ahr
- Figure S6 PFOS promotes IFN-γ production from both non-T and T cells *in vitro*
- Figure S7 Long-term PFOS treatment promotes production of IL-22 from both ILC3 and Th17 cells under the steady state
- Figure S8 PFOS affects the expression of RELM-β and mucin 2 under the steady state
- Supplemental figure legends
- Table S1. Primers used for Realtime RT-PCR
- Table S2 16S rRNA gene specific primers used in this study
- Supplemental References

Figure S1

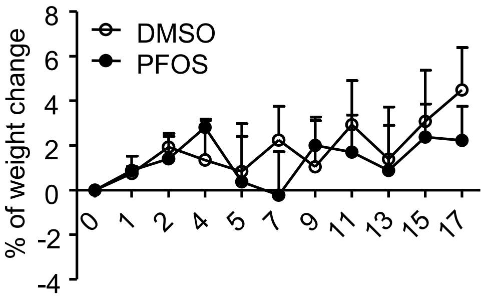

Figure S2

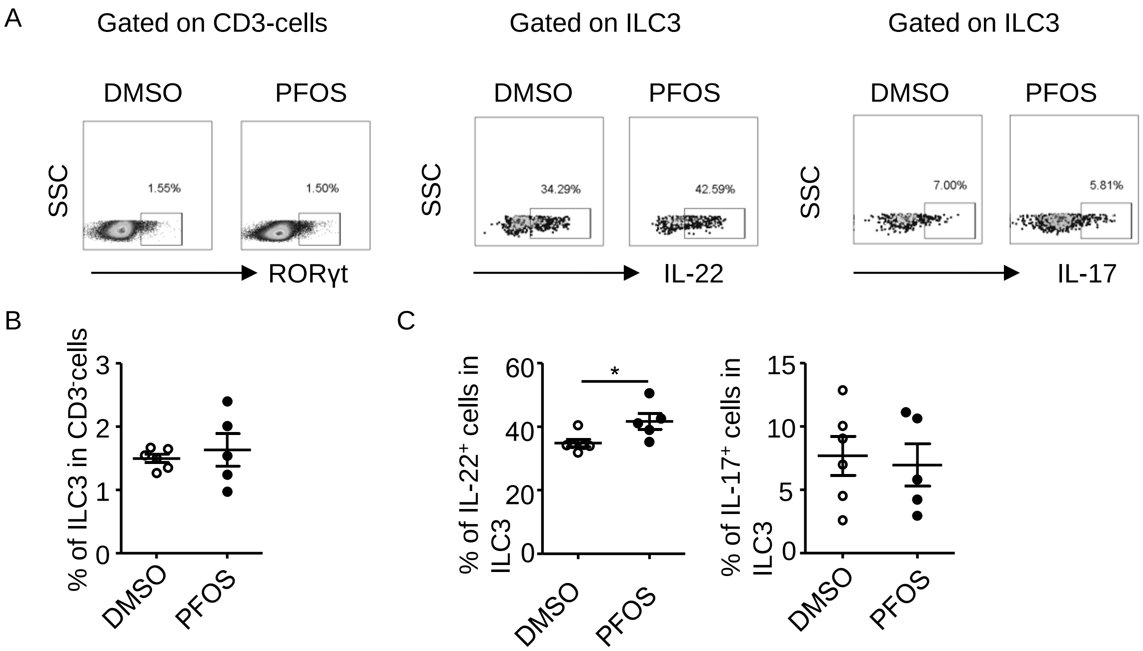

A
Gated on CD3-cells
Gated on ILC3
Gated on ILC3
DMSO
PFOS
DMSO
PFOS
DMSO
PFOS
SSC
1.55%
1.50%
34.29%
42.59%
7.00%
5.81%
RORγt
IL-22
IL-17
B
% of ILC3 in CD3-cells
C
*
% of IL-22+ cells in ILC3
% of IL-17+ cells in ILC3
DMSO
PFOS

Figure S3

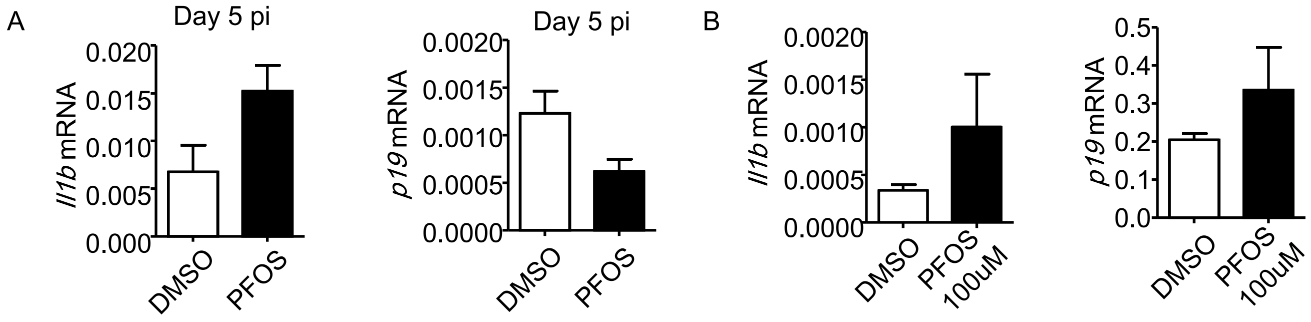

A
Day 5 pi
Il1b mRNA
0.020
0.015
0.010
0.005
0.000
DMSO
PFOS
Day 5 pi
p19 mRNA
0.0020
0.0015
0.0010
0.0005
0.0000
DMSO
PFOS
B
Il1b mRNA
0.0020
0.0015
0.0010
0.0005
0.0000
DMSO
PFOS 100uM
p19 mRNA
0.5
0.4
0.3
0.2
0.1
0.0
DMSO
PFOS 100uM

Figure S4

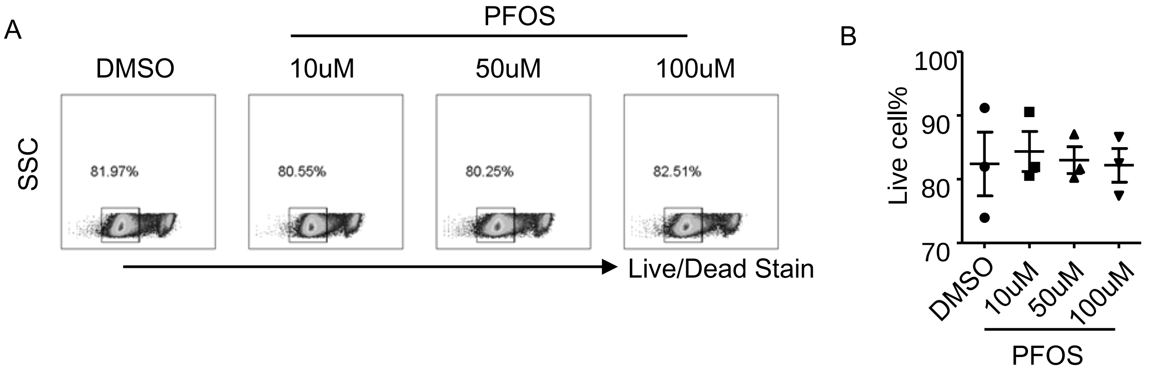
A
PFOS
DMSO
10uM
50uM
100uM
SSC
81.97%
80.55%
80.25%
82.51%
Live/Dead Stain
B
Live cell%
100
90
80
70
DMSO
10uM
50uM
100uM
PFOS

Figure S5

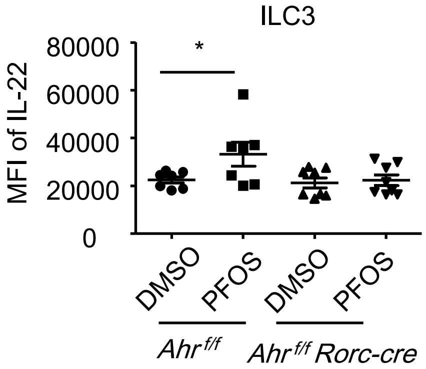

ILC3
MFI of IL-22
80000
60000
40000
20000
0
*
DMSO
PFOS
DMSO
PFOS
Ahr f/f
Ahr f/f Rorc-cre

Figure S6

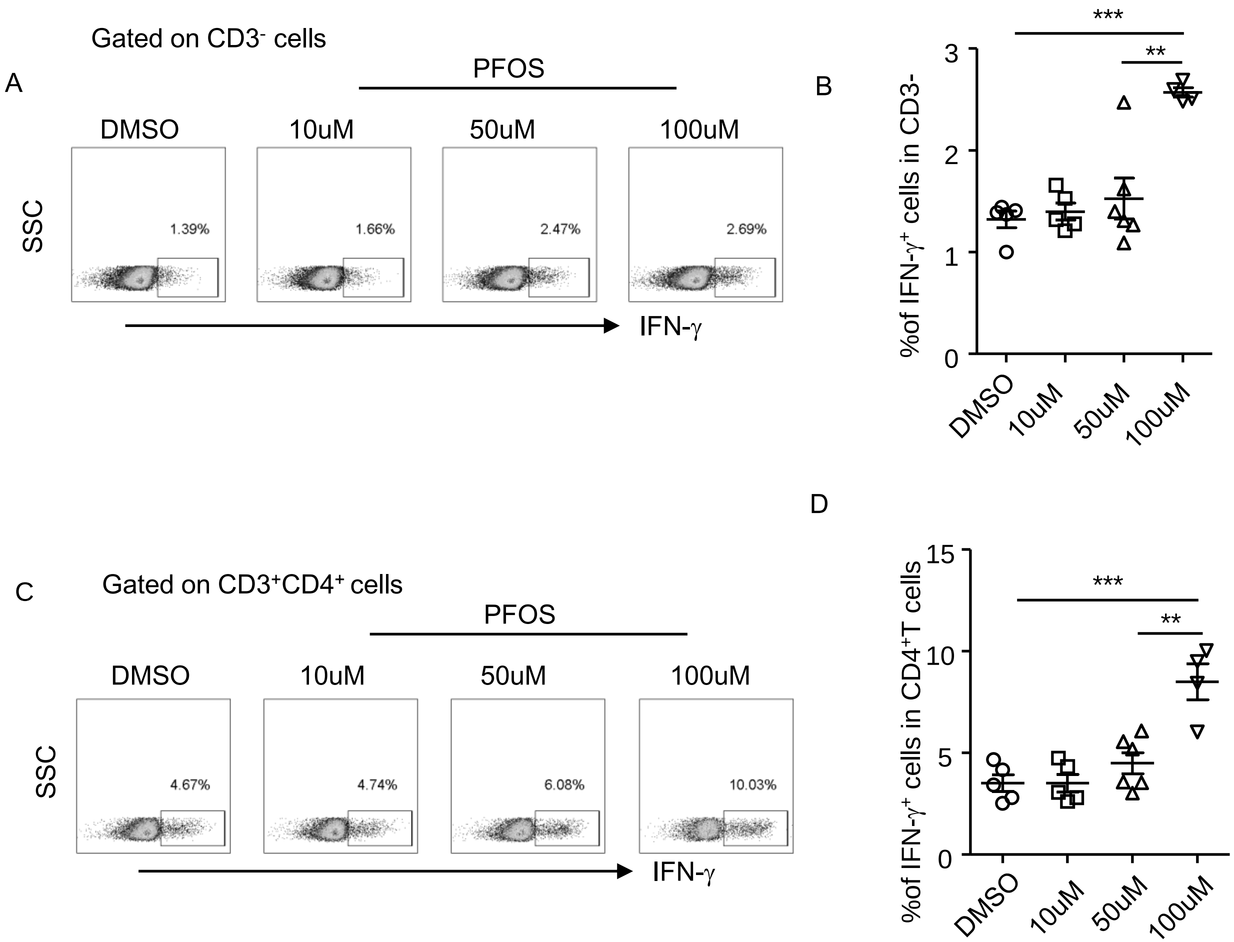
A
Gated on CD3- cells
PFOS
DMSO
10uM
50uM
100uM
SSC
1.39%
1.66%
2.47%
2.69%
IFN-γ
B
%of IFN-γ+ cells in CD3-
***
**
3
2
1
0
DMSO
10uM
50uM
100uM
C
Gated on CD3+CD4+ cells
PFOS
DMSO
10uM
50uM
100uM
SSC
4.67%
4.74%
6.08%
10.03%
IFN-γ
D
%of IFN-γ+ cells in CD4+T cells
***
**
15
10
5
0
DMSO
10uM
50uM
100uM

Figure S7

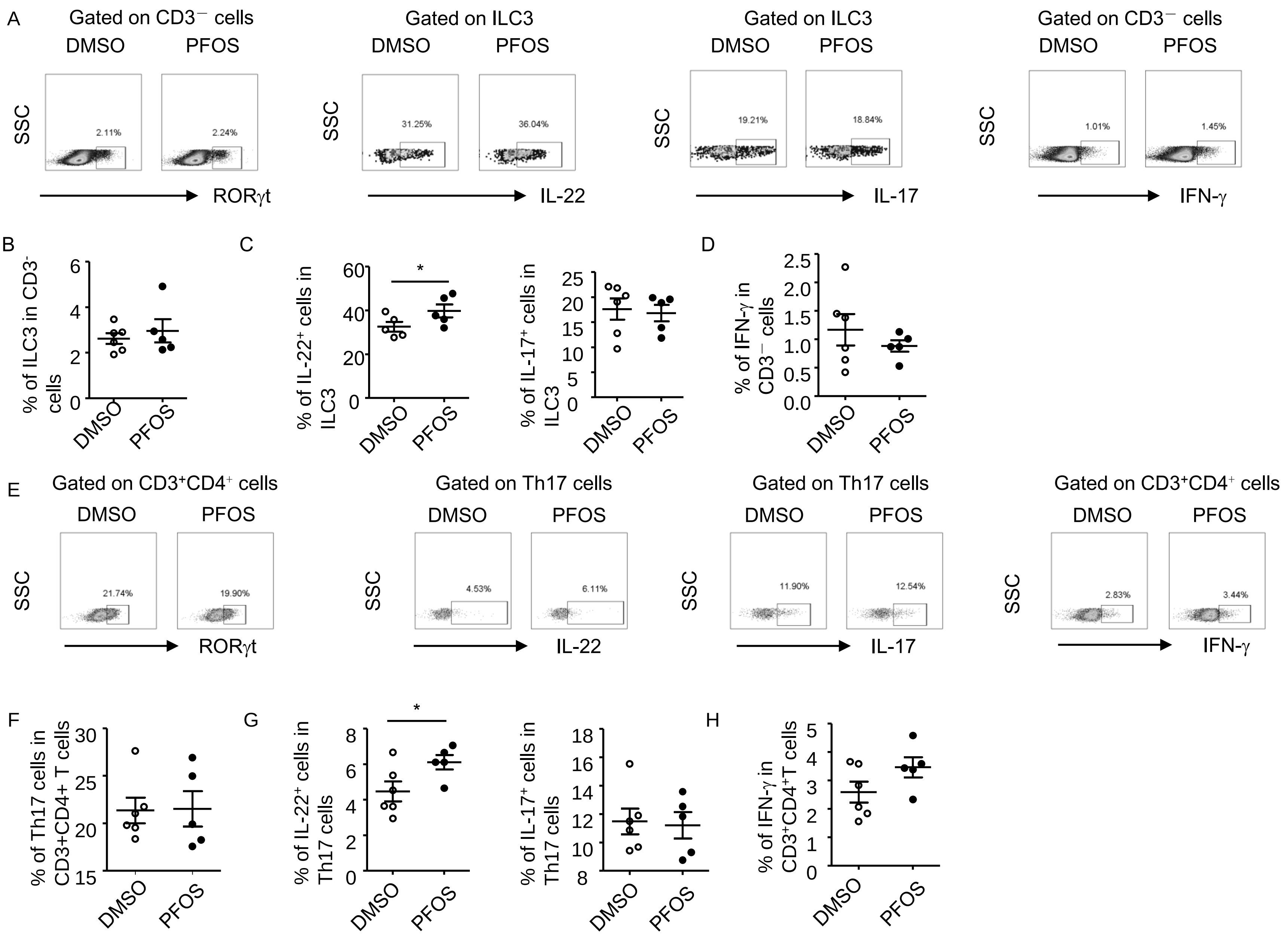
A
Gated on CD3⁻ cells
DMSO
PFOS
SSC
2.11%
2.24%
RORγt
Gated on ILC3
31.25%
36.04%
IL-22
19.21%
18.84%
IL-17
1.01%
1.45%
IFN-γ
B
% of ILC3 in CD3⁻ cells
C
% of IL-22⁺ cells in ILC3
*
% of IL-17⁺ cells in ILC3
D
% of IFN-γ in CD3⁻ cells
E
Gated on CD3⁺CD4⁺ cells
Gated on Th17 cells
21.74%
19.90%
4.53%
6.11%
11.90%
12.54%
2.83%
3.44%
F
% of Th17 cells in CD3+CD4+ T cells
G
% of IL-22⁺ cells in Th17 cells
% of IL-17⁺ cells in Th17 cells
H
% of IFN-γ in CD3⁺CD4⁺T cells

Figure S8

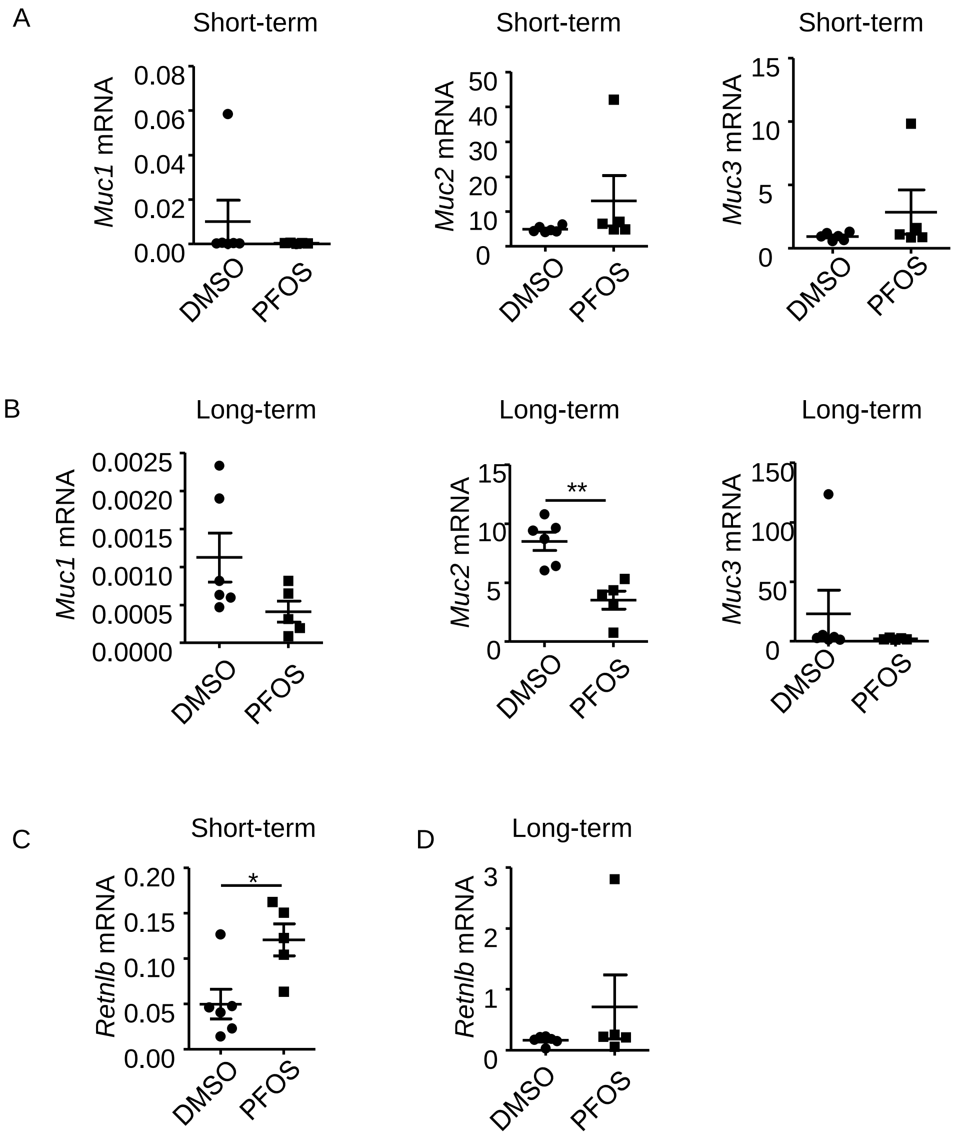
A
Short-term
Muc1 mRNA
0.08
0.06
0.04
0.02
0.00
DMSO
PFOS
Short-term
Muc2 mRNA
50
40
30
20
10
0
DMSO
PFOS
Short-term
Muc3 mRNA
15
10
5
0
DMSO
PFOS
B
Long-term
Muc1 mRNA
0.0025
0.0020
0.0015
0.0010
0.0005
0.0000
DMSO
PFOS
Long-term
Muc2 mRNA
15
10
5
0
**
DMSO
PFOS
Long-term
Muc3 mRNA
150
100
50
0
DMSO
PFOS
C
Short-term
Retnlb mRNA
0.20
0.15
0.10
0.05
0.00
*
DMSO
PFOS
D
Long-term
Retnlb mRNA
3
2
1
0
DMSO
PFOS

Figure S1 PFOS has no effect on change of body weight at steady state

Wild-type mice were treated by oral gavage with DMSO or PFOS (2mg/kg) in water on a daily basis. Percentages of weight change of the two groups of mice at indicated time points were shown. Data were pooled from 5 mice from each group. Error bars represent SEM. Data are representative of two independent experiments.

Figure S2 Short-term treatment of PFOS promotes the IL-22 production from ILC3 under the steady state

Wild-type mice were treated by oral gavage with DMSO or PFOS (2mg/kg) in water on a daily basis for 11 days. On day 12, large intestinal lamina proprial lymphocytes were isolated from each group and analyzed by flow cytometry. Cells were stimulated with PMA and ionomycin for 4 hr before analysis. (A) The expression of CD3, CD4, RORγt, IL-17, IL-22 was analyzed by flow cytometry. (B) Percentages of ILC3 ($CD3^-ROR\gamma t^+$ cells) gated on $CD3^-$ cells were shown. (C) Percentages of IL-22 and IL-17 expression gated on ILC3 were shown. Horizontal lines show the mean. Error bars represent SEM. Data are representative of two independent experiments.

Figure S3 PFOS has no impact on IL-1β and IL-23 mRNA expression

(A) On day 5 post *C. Rodentium* infection, large intestinal lamina proprial lymphocytes (LPL) were isolated from each group. The expression of *I11β* and *Il23a* (*p19*) in large intestinal LPLs was analyzed by realtime RT-PCR. (B) Large intestinal LPLs from *wild-type* mice were treated with PFOS at 100uM or DMSO for 20 hr. The expression of *I11β* and *Il23a* (*p19*) in large intestinal LPLs was analyzed by realtime RT-PCR. Error bars represent SEM. Data were pooled from three sets of experiments.

Figure S4 PFOS has no cytotoxic effect *in vitro*

Large intestinal LPLs were isolated from *wild-type* mice and treated with indicated concentrations of PFOS or DMSO for 20 hr. (A) Percentage of live cells was analyzed by staining with

“Fixable Violet Dead Cell Stain Kit” followed by flow cytometry. (B) Percentages of live cells in control or PFOS treated cells were shown. Error bars represent SEM. Data are representative of three independent experiments.

Figure S5 PFOS promotes IL-22 production from ILC3 on a per-cell-based level *in vitro* through Ahr

Large intestinal LPLs were isolated from $Ahr^{f/f}$*RORc-cre* or $Ahr^{f/f}$ mice and treated with PFOS at 100uM or DMSO for 20 hr. Cells were stimulated with PMA and ionomycin for the last 4 hr before harvested for flow cytometry analysis. Mean fluorescence intensity (MFI) of IL-22 gated on ILC3 from indicated groups was shown. Horizontal lines show the mean. Error bars represent SEM. Data were pooled from two independent experiments.

Figure S6 PFOS promotes IFN-γ production from both non-T and T cells *in vitro*

Large intestinal LPLs were isolated from wild-type mice and treated with indicated concentrations of PFOS or DMSO for 20 hr. Cells were stimulated with PMA and ionomycin for the last 4h before harvested for flow cytometry analysis. (A and C) The expression of CD3, CD4 and IFN-γ from indicated samples was analyzed by flow cytometry. (B) Percentages of IFN-γ expression gated on $CD3^-$ were shown. (D) Percentages of IFN-γ expression gated on $CD3^+CD4^+$ cells were shown. Horizontal lines show the mean. Error bars represent SEM. Data are representative of two independent experiments.

Figure S7 Long-term PFOS treatment promotes production of IL-22 from both ILC3 and Th17 cells under the steady state

Wild-type mice were treated by oral gavage with DMSO or PFOS (2mg/kg) in water on a daily basis for 17 days. On day 18, large intestinal lamina proprial lymphocytes were isolated from each group and analyzed by flow cytometry. Cells were stimulated with PMA and ionomycin for 4 hr before analysis. (A and E) The expression of CD3, CD4, RORγt, IL-17, IL-22 and

IFN-γ was analyzed by flow cytometry. (B) Percentages of ILC3 ($CD3^-ROR\gamma t^+$ cells) gated on $CD3^-$ cells were shown. Statistical analyses were performed using paired t-test. (C) Percentages of IL-22 and IL-17 expression gated on ILC3 were shown. (D) Percentages of IFN-γ gated on $CD3^-$ cells were shown. (F) Percentages of Th17 cells ($CD3^+CD4^+ROR\gamma t^+$ cells) gated on $CD3^+CD4^+$ cells were shown. (G) Percentages of IL-22 and IL-17 production gated on Th17 cells were shown. (H) Percentages of IFN-γ gated on $CD3^+CD4^+$ cells were shown. Horizontal lines show the mean. Error bars represent SEM. Data are representative of two independent experiments.

Figure S8 PFOS affects the expression of RELM-β and mucin 2 under the steady state

Wild-type mice were treated by oral gavage with DMSO or PFOS (2mg/kg) in water on a daily basis for 11(short-term) or 17(long-term) days. (A) mRNA expression of Muc1, Muc2 and Muc3 on day 12 post PFOS treatment in colon tissues was analyzed by realtime RT-PCR. (B) mRNA expression of Muc1, Muc2 and Muc3 on day18 post PFOS treatment in colon tissues was analyzed by realtime RT-PCR. (C) mRNA expression of *Retnlb* on day 12 post PFOS treatment in colon tissues was analyzed by realtime RT-PCR. (D) mRNA expression of *Retnlb* on day 18 post PFOS treatment in colon tissues was analyzed by realtime RT-PCR. Horizontal lines show the mean. Error bars represent SEM. Data were pooled from two independent experiments.

Table S1. Primers used for Realtime RT-PCR

**Gene**	**Primer**
Cyp1a1-FW	5'- GTCCCTCCTTACAGCCCAAG-3'
Cyp1a1-RV	5'- GCTCTGACCACCCAGAATCC-3'
p19-FW	5'-CCAGTTCTGCTTGCAAAGG-3'
p19-RV	5'-GGTGATCCTCTGGCTGGA -3'
Il1β-FW	5'-TGCCACCTTTTGACAGTGATG-3'
Il1β -RV	5'-TGATGTGCTGCTGCGAGATT-3'
Muc1-FW	5'-GGAACATTTCTGGATTGTTTCTGC-3'
Muc1- RV	5'-ACTGCATCTCATTCACTTTGACT-3'
Muc2-FW	5'-GCTGACGAGTGGTTGGTGAATG-3'
Muc2- RV	5'-GATGAGGTGGCAGACAGGAGAC-3'
Muc3-FW	5'-GGAACTGGTGGAGAGCGTAG-3'
Muc3- RV	5'-GGTGTACTTGGCCTTCAGGA-3'
Retnlb-FW	5'-TTTCCTTTTCATCCTCGTCTCCC-3'
Retnlb-RV	5'-GTGACAACCATCCCAGCAGGA-3'
Reg3b-FW	5'-CCACTCTGGGTGCAGAAC-3'
Reg3b-RV	5'-AATTCGGGATGTTTGCTGTC-3'
Reg3g-FW	5'-CAAGGTGAAGTTGCCAAGAA-3'
Reg3g-RV	5'-CCTCTGTTGGGTTCATAGCC-3'

Table S2 16S rRNA gene specific primers used in this study

Group	Taxonomic rank	Primer	Sequence(5'to 3')
Bacteria [1]	Order	UniF340	ACTCCTACGGGAGGCAGCAGT
		UniR514	ATTACCGCGGCTGCTGGC
Actinobacteria [2]	Phylum	Act920F3	TACGGCCGCAAGGCTA
		Act1200R	TCRTCCCCACCTTCCTCCG
Bacteroidetes [3]	Phylum	Bact934F	GGARCATGTGGTTTAATTCGATGAT
		Bact1060R	AGCTGACGACAACCATGCAG
Firmicutes [4]	Phylum	928F-Firm	TGAAACTYAAAGGAATTGACG
		1040FirmR	ACCATGCACCACCTGTC
Proteobacteria [5]	Phylum	Proteobacteria-F	CATGACGTTACCCGCAGAAGAAG
		Proteobacteria-R	CTCTACGAGACTCAAGCTTGC
Bacteroides [1]	Genus	BactF285	GGTTCTGAGAGGAGGTCCC
		UniR338	GCTGCCTCCCGTAGGAGT
Lactobacillus [1]	Genus	LabF362	AGCAGTAGGGAATCTTCCA
		LabR677	CACCGCTACACATGGAG
Enterbacteriaceae [1]	Family	Uni515F	GTGCCAGCAGCCGCGGTAA
		Ent826R	GCCTCAAGGGCACAACCTCCAAG
Clostridium coccoides [1]	Species	UniF338	ACTCCTACGGGAGGCAGC
		C.cocR491	GCTTCTTAGTCAGGTACCGTCAT

E.coli [6]	Species	E.coli-F	CATGCCGCGTGTATGAAGAA
		E.coli-R	CGGGTAACGTCAATGAGCAAA
Lactobacillus acidophilus [7]	Species	F_acid	GAAAGAGCCCAAACCAAGTGATT
		R_acid	CTTCCCAGATAATTCAACTATCGCTTA
Lactobacillus casei [7]	Species	F_casei	CTATAAGTAAGCTTTGATCCGGAGATT T
		R_casei	CTTCCTGCGGGTACTGAGATGT
Lactobacillus johnsonni [8]	Species	Lj1	CACTAGACGCATGTCTAGAG
		La2	AGTCTCTCAACTCGGCTATG
Lactobacillus reuteri [7]	Species	F_reut	ACCGAGAACACCGCGTTATTT
		R_reut	CATAACTTAACCTAAACAATCAAAGAT TGTCT

References

1. Barman, M., et al., *Enteric salmonellosis disrupts the microbial ecology of the murine gastrointestinal tract.* Infect Immun, 2008. **76**(3): p. 907-15.
2. Larmonier, C.B., et al., *Reduced colonic microbial diversity is associated with colitis in NHE3-deficient mice.* Am J Physiol Gastrointest Liver Physiol, 2013. **305**(10): p. G667-77.
3. Guo, X., et al., *Development of a real-time PCR method for Firmicutes and Bacteroidetes in faeces and its application to quantify intestinal population of obese and lean pigs.* Lett Appl Microbiol, 2008. **47**(5): p. 367-73.
4. Pedersen, R., et al., *Changes in the gut microbiota of cloned and non-cloned control pigs during development of obesity: gut microbiota during development of obesity in cloned pigs.* BMC Microbiol, 2013. **13**: p. 30.
5. Queipo-Ortuno, M.I., et al., *Influence of red wine polyphenols and ethanol on the gut microbiota ecology and biochemical biomarkers.* Am J Clin Nutr, 2012. **95**(6): p. 1323-34.
6. Arthur, J.C., et al., *Intestinal inflammation targets cancer-inducing activity of the microbiota.* Science, 2012. **338**(6103): p. 120-3.
7. Cui, Y., et al., *Different Effects of Three Selected Lactobacillus Strains in Dextran Sulfate Sodium-Induced Colitis in BALB/c Mice.* PLoS One, 2016. **11**(2): p.

e0148241.
8. Furet, J.P., P. Quenee, and P. Tailliez, *Molecular quantification of lactic acid bacteria in fermented milk products using real-time quantitative PCR.* Int J Food Microbiol, 2004. **97**(2): p. 197-207.
